# Endothelial, cytokine, and Th1/cellular host biomarkers discriminate complicated osteoarticular tuberculosis: a prospective single-center cohort study with ROC analysis

**DOI:** 10.3389/fcimb.2026.1866153

**Published:** 2026-05-29

**Authors:** Shuhrat Bozorov, Bakhtiyor Khamdamov, Isomiddin Usmanov, Nigora Faizullaeva, Musharraf Shodiyeva

**Affiliations:** 1 Bukhara State Medical Institute, Bukhara, Uzbekistan; 2 Institute of Immunology and Human Genomics of the Academy of Sciences of the Republic of Uzbekistan, Tashkent, Uzbekistan

**Keywords:** endothelial dysfunction, extrapulmonary tuberculosis, host immunological biomarkers, interferon-gamma, osteoarticular tuberculosis, prognostic stratification, ROC analysis, sICAM-1

## Abstract

**Background:**

Osteoarticular tuberculosis (OATB) accounts for 10–15% of extrapulmonary TB cases and causes progressive bone destruction, neurological compromise, and persistent disability. No validated host-based framework currently identifies patients at high risk of complicated disease course at baseline, and the integrated role of cellular, cytokine, endothelial, and fibrinolytic axes in OATB has not been systematically characterized.

**Methods:**

We conducted a prospective single-center observational study at Bukhara Regional Center of Phthisiopulmonology, Uzbekistan. Of 223 consecutive eligible adults with confirmed OATB, 115 met immunological-cohort criteria, with peripheral blood drawn before anti-tuberculosis chemotherapy. *A priori* panel of 20 markers was quantified by flow cytometry and ELISA, covering lymphocyte subpopulations, cytokines, endothelial markers, fibrinolytic markers, and immunoglobulins. Pairwise correlation and ROC analyses with Youden-*J* thresholding were performed against complicated versus uncomplicated course in 30 prognostic-subset patients.

**Results:**

Nine markers achieved high prognostic discrimination: sICAM-1 (0.89), IL-6 (0.88), sVCAM-1 (0.87), CD4/CD8 (0.86), IP-10 (0.85), TNF-α (0.84), CD95^+^ (0.83), IFN-γ (0.82), and PAI-1 (0.81), with sensitivities of 76.7–86.7% and specificities of 73.3–83.3%. These markers mapped onto three coupled pathophysiological axes — endothelial-fibrinolytic, pro-inflammatory cytokine, and Th1/cellular with apoptotic priming converging in a plausible immunopathological pattern in complicated disease.

**Conclusions:**

Nine individually high-performing baseline markers discriminate complicated from uncomplicated OATB with clinically useful accuracy, with endothelial-fibrinolytic markers contributing the highest AUC values. External validation in multi-center cohorts is the essential next step toward routine OATB risk stratification.

## 1 Introduction

Tuberculosis (TB) remains the leading cause of death from a single infectious agent worldwide, with an estimated 10.8 million people developing TB and 1.25 million dying of the disease in 2023; among 8.2 million people newly diagnosed and notified, 16% had extrapulmonary tuberculosis (EPTB) (World Health Organization, 2024). Within EPTB, osteoarticular tuberculosis (OATB) — comprising spinal involvement (Pott disease), large-joint disease (hip, knee), and pelvic and extremity disease accounts for approximately 10–15% of cases, with spinal involvement representing roughly half of all musculoskeletal TB (Leowattana et al., 2023; Kumari et al., 2025). Recent multi-center cohorts confirm that OATB is rising in both high-burden and low-burden settings, driven by drug resistance, comorbidity, and global migration (Fang et al., 2024; Yadav et al., 2023).

Despite multidrug chemotherapy that cures most patients with drug-susceptible disease, OATB carries a disproportionate clinical impact: progressive vertebral collapse, paraspinal abscess, and neurological compromise are well-described complications, particularly when diagnosis is delayed (Glassman et al., 2023; Leowattana et al., 2023). Conventional inflammatory markers such as ESR and CRP, although routinely used, lack the specificity required for prognostic stratification in OATB and may remain normal even in active disease (Glassman et al., 2023). No validated framework currently exists to identify patients at high risk of complicated outcome before complications occur, and the immunopathological basis for differential disease trajectories in OATB remains poorly characterized.

The host immune response to *Mycobacterium tuberculosis* is increasingly recognized as a determinant of disease trajectory. The classical Th1 paradigm — protective IFN-γ-driven macrophage activation orchestrated by CD4^+^ T cells is well established in pulmonary TB and underpins IGRA-based diagnostics (Pai et al., 2016), and the IFN-γ-induced chemokine IP-10/CXCL10 has emerged as a clinically useful biomarker of mycobacterial antigen recognition (Strzelak et al., 2024; Khalid et al., 2023). More recent work has highlighted three additional axes that shape outcome in pulmonary TB: pro-inflammatory cytokine excess (IL-6, TNF-α, IL-1β), where elevated baseline IL-6 has been validated as a biomarker for unfavorable treatment outcomes (Sampath et al., 2023; Gupte et al., 2022); Fas-mediated apoptotic priming of lymphocytes (CD95/Fas), coupling chronic antigenic stimulation to effector-cell exhaustion (Salina and Morozova, 2015); and microvascular endothelial activation reflected in soluble adhesion molecules (sICAM-1, sVCAM-1) and dysregulated fibrinolysis (Hamzaoui et al., 1996; Singh et al., 2023). Cytokine-based indicators have also been applied to identify the transition between disease states in other chronic inflammatory conditions in our region (Shodieva et al., 2024). However, how these axes interact specifically in OATB, where avascular cortical bone, sequestered necrotic tissue, and limited drug penetration create conditions favoring mycobacterial persistence has not been systematically addressed. Existing OATB biomarker work has focused predominantly on diagnosis rather than prognosis (Chen et al., 2022).

We therefore tested the hypothesis that complicated OATB is characterized by a coordinated, measurable shift from Th1-dominant cellular control toward endothelial activation, pro-inflammatory cytokine excess, and apoptosis-prone lymphocyte phenotypes and that a defined subset of host markers, measured in peripheral blood at baseline before anti-tuberculosis chemotherapy, would discriminate patients at high risk of complicated outcome. To address this, we prospectively quantified an *a priori* panel of 20 cellular, humoral, cytokine, endothelial, and fibrinolytic markers in 115 adults with confirmed OATB at a regional phthisiopulmonology center in Uzbekistan, and applied correlation and ROC analyses to identify markers and cut-offs that separate complicated from uncomplicated disease course.

## 2 Methods

### 2.1 Study design and setting

This was a prospective observational study conducted at the Bukhara Regional Center of Phthisiopulmonology (Bukhara, Uzbekistan) between January 2022 and December 2024. The protocol was approved by the Ethics Committee of Bukhara State Medical Institute and conducted in accordance with the Declaration of Helsinki. All participants provided written informed consent.

### 2.2 Participants and study cohorts

Adults (≥ 18 years) with an established diagnosis of osteoarticular tuberculosis, based on combined clinical, radiological (chest radiography and multi-slice CT/MRI of the affected segment), and laboratory findings, were eligible. Exclusion criteria were acute myocardial infarction, acute renal or hepatic failure, pregnancy or lactation, and active malignancy.

Of 223 consecutive eligible patients, 115 met the additional criteria for the immunological cohort: (i) no prior corticosteroid or cytostatic therapy, and (ii) peripheral venous blood drawn on the day of admission, before initiation of anti-tuberculosis chemotherapy. All patients subsequently received standard four-drug intensive-phase therapy — isoniazid, rifampicin, pyrazinamide, and ethambutol in fixed-dose combinations (H75/R150/Z400/E275) for 56 doses, followed by an isoniazid–rifampicin (H75/R150) continuation phase for 112 doses, in accordance with the National Tuberculosis Programme of Uzbekistan and WHO Category I recommendations.

Within the immunological cohort, a prognostic subset of 30 patients with complicated course of OATB was identified for correlation and ROC analyses; the remaining patients of the same cohort with uncomplicated course served as the comparator group. Complicated course was defined *a priori* as the presence of one or more of: (i) neurological complications (spinal cord compression, myelopathy, paraparesis or paraplegia, or pelvic-organ dysfunction); (ii) purulent-destructive complications (paravertebral or epidural abscess, epiduritis, or fistula formation); or (iii) progressive orthopedic complications (coxarthrosis or gonarthrosis grade II–III, joint deformity, or prosthesis instability).

Outcome assessment was performed independently of baseline immunological data. The classification of patients into complicated and uncomplicated courses was based solely on clinical, radiological, and surgical criteria documented in the medical records by the treating clinical team (orthopedic surgeon, phthisiatrician, and neurologist) during the inpatient treatment period. Importantly, the immunological marker panel was processed and analyzed in a separate immunological laboratory, and the results were not available to the treating clinicians at the time of outcome classification, as blood samples were stored and analyzed in batches after the clinical follow-up period. This temporal and organizational separation between laboratory analysis and clinical outcome assessment effectively established operational blinding and eliminated the possibility of differential misclassification bias.

Outcome classification was not performed by a single rater. According to the standard multidisciplinary protocol at the Bukhara Regional Center for Phthisiology and Pulmonology, the designation of a complicated course was determined by a clinical consultant consisting of a treating phthisiologist, an orthopedic surgeon, and a neurologist based on documented clinical findings at the time of discharge from the hospital. The follow-up window was standardized across patients: all outcomes were assessed after the completion of the intensive phase of inpatient treatment (56 days/56 doses of HRZE regimen), and complications were recorded during the hospitalization period. Therefore, the follow-up for outcome assessment was uniform and corresponded to the period of inpatient treatment (mean duration 60 ± 8 days). Formal inter-rater reliability tests (e.g., Cohen’s kappa) were not performed because the classification of outcomes was based on consensus of the multidisciplinary panel rather than independent parallel assessments; this was recognized as a limitation.

### 2.3 Immunological measurements

Flow cytometry was performed on a FACSCalibur platform (BD Biosciences) using fluorochrome-conjugated monoclonal antibodies (anti-CD3-FITC, anti-CD4-PE, anti-CD8-PerCP, anti-CD16-PE, anti-CD20-FITC, anti-CD25-PE, anti-CD95-FITC; BD Biosciences). Lymphocytes were gated by forward/side scatter, and a minimum of 10,000 events were acquired per sample. Cytokine and endothelial marker concentrations were determined by sandwich ELISA using commercial kits (Vector-Best, Russia) according to the manufacturer’s instructions, with intra-assay CV < 8% and inter-assay CV < 12%. All samples were run in duplicate.

At baseline, peripheral venous blood was assayed for: (i) lymphocyte subpopulations CD3^+^, CD4^+^, CD8^+^, CD16^+^, CD20^+^, CD25^+^, and CD95^+^ by flow cytometry, with computation of the CD4/CD8 ratio; (ii) serum cytokines IL-1β, IL-4, IL-6, TNF-α, IFN-γ, and IP-10/CXCL10; (iii) endothelial activation markers soluble ICAM-1 and VCAM-1; (iv) fibrinolytic markers tissue plasminogen activator (tPA) and plasminogen activator inhibitor-1 (PAI-1); and (v) immunoglobulins IgA, IgG, and IgM. All soluble markers were quantified by sandwich enzyme-linked immunosorbent assay (ELISA) using commercial kits (Vector-Best, Russian Federation; ElisaKit, China) strictly according to manufacturer instructions.

### 2.4 Statistical analysis

Continuous variables were tested for normality with the Shapiro–Wilk test and reported as mean ± SEM (parametric distributions) or median with interquartile range (non-parametric). Pairwise correlations across all measured immunological markers were computed using the Pearson coefficient (parametric distributions) or the Spearman rank coefficient (non-parametric); |r| ≥ 0.30 with p < 0.05 was considered structurally meaningful, in line with conventional Chaddock-scale thresholds. Receiver operating characteristic (ROC) analysis was performed for each marker against the binary outcome (complicated versus uncomplicated course); optimal cut-off thresholds were defined by maximization of Youden’s *J* statistic, and AUC values were classified as high (≥ 0.80), moderate (0.70–0.79), or low (< 0.70). All analyses were performed using Jamovi v2.7.9 and Microsoft Excel 16.0; two-tailed p < 0.05 was considered statistically significant.

## 3 Results

### 3.1 Cohort characteristics

**Figure 1:**
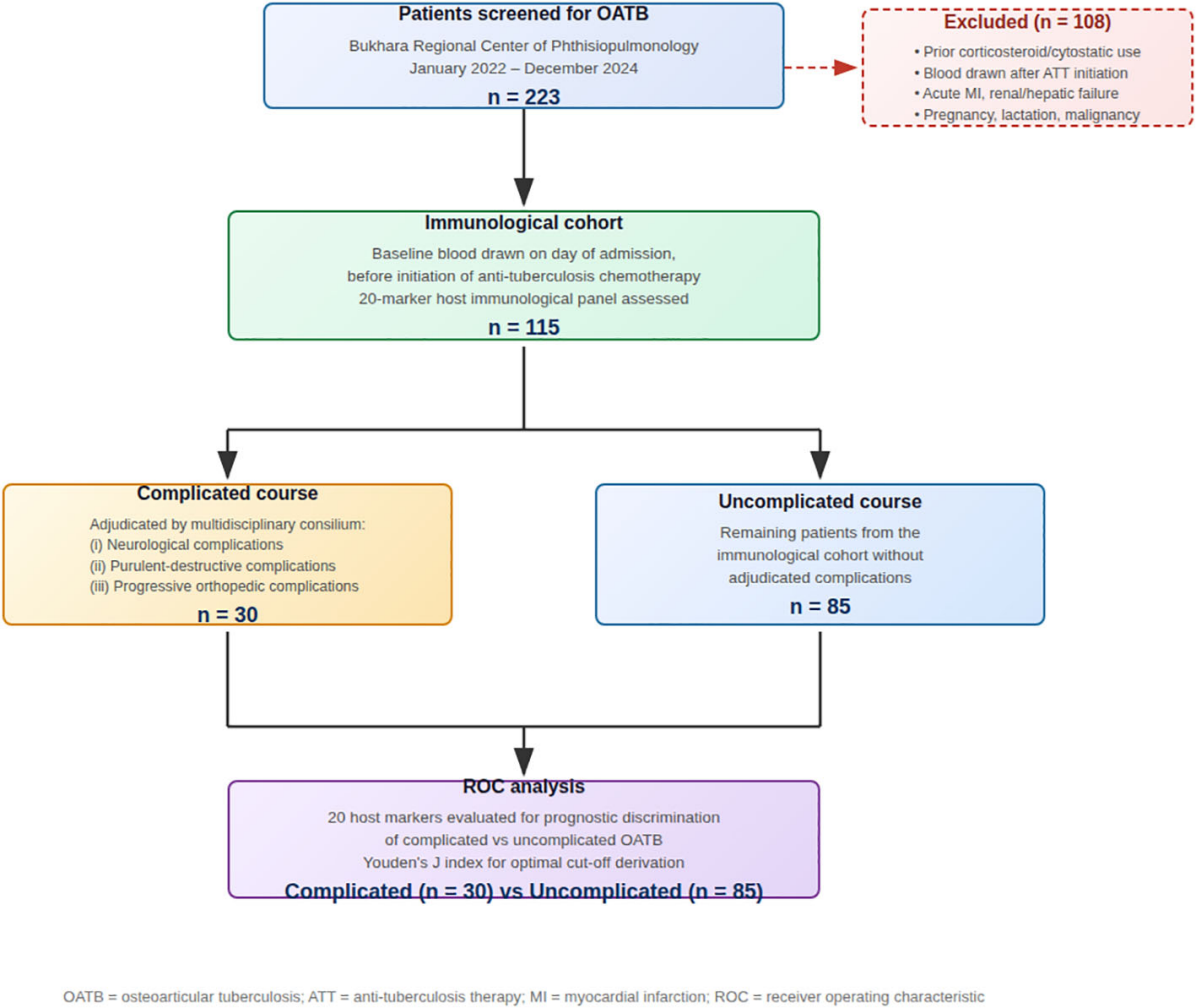
Participant flow diagram.

The immunological cohort comprised 115 adults with confirmed OATB, recruited from a screened population of 223 consecutive patients managed at Bukhara Regional Center of Phthisiopulmonology between January 2022 and December 2024. In the broader screened cohort the male-to-female ratio was 58.4%: 41.6%, with a mean age of 46.6 ± 2.1 years (range 18–87); the immunological subset (n = 115) was drawn consecutively from this source population without selection on demographic features and did not differ significantly from the parent cohort in age or sex distribution (p > 0.05). The prognostic subset (n = 30) followed for adjudicated outcome was demographically and clinically representative of the immunological cohort, with no significant differences in age, sex, or anatomical site of disease (p > 0.05 for all comparisons). Complicated course was adjudicated by a multidisciplinary consilium at the completion of the intensive inpatient treatment phase (56 days) (**Figure 1**).

**Table 1** presents the main clinical and demographic characteristics of the prognostic subgroup, stratified by outcome group. The complex group showed significantly more advanced histomorphological stage (late stages IV-V: 60.0% vs. 13.3%, p = 0.01), more frequent neurological deficits at admission (gait disturbance: 60.0% vs. 20.0%, p = 0.02), and higher baseline inflammatory markers (ESR: 42.3 vs. 28.6 mm/h, p < 0.01; CRP: 48.5 vs. 22.1 mg/L, p < 0.01). Diabetes mellitus was more common in the complex group (40.0% vs. 20.0%), but this difference was not statistically significant (p = 0.23). The groups did not differ significantly in age, gender, anatomical location, or cardiovascular disease.

**Table 1:** Baseline clinical and demographic characteristics of the prognostic subset, stratified by outcome group (n = 30).

Characteristic	Complicated (n=15)	Uncomplicated (n=15)	p-value
Demographics			
Age, years (mean ± SD)	49.2 ± 11.3	44.1 ± 10.8	0.21
Male sex, n (%)	9 (60.0)	8 (53.3)	0.71
Disease duration			
Symptom duration < 1 year, n (%)	1 (6.7)	5 (33.3)	0.07
Symptom duration 1–5 years, n (%)	6 (40.0)	7 (46.7)	0.71
Symptom duration > 5 years, n (%)	8 (53.3)	3 (20.0)	0.05
Anatomical site			
Spinal (thoracic/lumbar/cervical), n (%)	10 (66.7)	8 (53.3)	0.46
Hip/knee (large joint), n (%)	3 (20.0)	5 (33.3)	0.41
Pelvic/extremity, n (%)	2 (13.3)	2 (13.3)	1.00
Histomorphological stage			
Early (stage I–II), n (%)	1 (6.7)	6 (40.0)	0.03*
Intermediate (stage II–III), n (%)	5 (33.3)	7 (46.7)	0.46
Late (stage IV–V), n (%)	9 (60.0)	2 (13.3)	0.01*
Neurological status at admission			
Gait disturbance/limping, n (%)	9 (60.0)	3 (20.0)	0.02*
Urinary/fecal incontinence, n (%)	4 (26.7)	0 (0.0)	0.03*
Comorbidities			
Diabetes mellitus type 2, n (%)	6 (40.0)	3 (20.0)	0.23
Cardiovascular disease (HTN/IHD), n (%)	8 (53.3)	7 (46.7)	0.72
Chronic respiratory disease, n (%)	4 (26.7)	2 (13.3)	0.36
Renal disease, n (%)	4 (26.7)	2 (13.3)	0.36
Laboratory at baseline			
ESR, mm/h (mean ± SD)	42.3 ± 12.1	28.6 ± 9.4	<0.01*
CRP, mg/L (mean ± SD)	48.5 ± 18.3	22.1 ± 11.2	<0.01*
Hemoglobin, g/L (mean ± SD)	96.3 ± 11.8	112.5 ± 10.2	<0.01*

Data presented as mean ± SD or n (%). Comparisons between groups: Student’s t-test for continuous variables, Fisher’s exact test for categorical variables. HTN, hypertension; IHD, ischemic heart disease. *p < 0.05.

### 3.2 Correlation structure of immune dysregulation

Pairwise correlation analysis across the 20-marker panel (n = 115) revealed a coordinated immunopathological network organized around three coupled sub-clusters.

The Th1 axis showed the strongest internal coupling. The CD4/CD8 ratio correlated positively with IFN-γ (r = 0.73, p < 0.001) and IP-10/CXCL10 (r = 0.72, p < 0.001); CD4^+^ T-cell percentage correlated positively with IFN-γ (r = 0.39) and IP-10 (r = 0.35; both p < 0.01). Reciprocally, CD8^+^ T-cell percentage correlated negatively with IFN-γ (r = −0.43) and IP-10 (r = −0.45; both p < 0.01).

The pro-inflammatory cytokine cluster was tightly co-activated: TNF-α with IL-6 (r = 0.42), TNF-α with IL-1β (r = 0.46), and IL-6 with IL-1β (r = 0.43); all p < 0.01. Cross-axis coupling linked this cluster to the vascular compartment (sICAM-1 and sVCAM-1 with pro-inflammatory cytokines, r = 0.14–0.25, p < 0.05) and to the fibrinolytic compartment, where PAI-1 correlated positively with immunoregulatory imbalance (r = 0.20–0.25) and tPA correlated negatively with endothelial and inflammatory markers (r = −0.15 to −0.20); both p < 0.05.

In the complicated-course subset the network was hyper-coupled: IgG with IL-1β (r = 0.69, p < 0.001) and IL-6 (r = 0.60, p < 0.01); CD25^+^ and CD95^+^ with TNF-α and IL-6 (r = 0.42–0.56, p < 0.01); a tight TNF-α–IL-6–IL-1β triangle (r = 0.65–0.78, p < 0.001); and an inverse tPA–PAI-1 relationship (r = −0.39, p < 0.05) reflecting suppressed fibrinolytic activity. Collectively, these patterns indicate convergence of the pro-inflammatory, humoral, and apoptosis-priming axes as disease progresses toward complication.

### 3.3 ROC analysis: prognostic discrimination of complicated OATB

ROC analysis identified nine markers with high prognostic discrimination for complicated course (AUC ≥ 0.80, p < 0.01; **Table 2**). Endothelial activation markers ranked highest — sICAM-1 (AUC 0.89; cut-off ≥ 620 ng/mL) and sVCAM-1 (AUC 0.87; ≥ 735 ng/mL) — followed by the pro-inflammatory cytokines IL-6 (AUC 0.88; ≥ 45 pg/mL) and TNF-α (AUC 0.84; ≥ 54 pg/mL). Markers of Th1/cellular immunity — the CD4/CD8 ratio (AUC 0.86; ≤ 0.90), IP-10 (0.85; ≤ 8.5 pg/mL), and IFN-γ (0.82; ≤ 10 pg/mL) — together with the apoptosis-priming marker CD95^+^ (0.83; ≥ 27%) and the fibrinolytic marker PAI-1 (0.81; ≥ 80 ng/mL) completed the top tier. Optimal thresholds achieved sensitivities of 76.7–86.7% and specificities of 73.3–83.3%.

**Table 2:** Receiver operating characteristic analysis of 20 host immunological markers for discrimination of complicated osteoarticular tuberculosis (n = 30).

Marker	AUC (95% CI)	Cut-off	Sens (%)	Spec (%)	p	Tier
sICAM-1 (ng/mL)	0.89 (0.76–0.97)	≥ 620	86.7	83.3	< 0.001	**High**
IL-6 (pg/mL)	0.88 (0.74–0.96)	≥ 45.0	86.7	83.3	< 0.001	**High**
sVCAM-1 (ng/mL)	0.87 (0.73–0.96)	≥ 735	83.3	80.0	< 0.001	**High**
CD4/CD8 ratio	0.86 (0.72–0.95)	≤ 0.90	83.3	80.0	< 0.001	**High**
IP-10 (pg/mL)	0.85 (0.70–0.94)	≤ 8.5	86.7	73.3	< 0.001	**High**
TNF-α (pg/mL)	0.84 (0.69–0.94)	≥ 54.0	83.3	80.0	< 0.001	**High**
CD95^+^ (%)	0.83 (0.68–0.93)	≥ 27	80.0	76.7	< 0.001	**High**
IFN-γ (pg/mL)	0.82 (0.67–0.93)	≤ 10.0	80.0	76.7	< 0.001	**High**
PAI-1 (ng/mL)	0.81 (0.66–0.92)	≥ 80	76.7	80.0	0.001	**High**
IgG (g/L)	0.79 (0.63–0.91)	≥ 15.5	76.7	73.3	0.002	Moderate
IL-1β (pg/mL)	0.66 (0.47–0.82)	≥ 14.0	63.3	60.0	0.08	Low
IL-4 (pg/mL)	0.64 (0.45–0.81)	≥ 23.0	60.0	56.7	0.11	Low
CD25^+^ (%)	0.63 (0.44–0.80)	≥ 23	60.0	63.3	0.10	Low
CD4^+^ (%)	0.62 (0.43–0.80)	≤ 27	60.0	63.3	0.09	Low
IgA (g/L)	0.61 (0.42–0.79)	≥ 2.9	56.7	60.0	0.14	Low
tPA (ng/mL)	0.60 (0.41–0.78)	≤ 6.0	56.7	63.3	0.15	Low
IgM (g/L)	0.59 (0.40–0.77)	≥ 1.6	53.3	56.7	0.18	Low
CD3^+^ (%)	0.58 (0.39–0.76)	≤ 49	56.7	56.7	0.21	Low
CD16^+^ (%)	0.58 (0.39–0.76)	≥ 24	53.3	56.7	0.22	Low
CD20^+^ (%)	0.56 (0.37–0.74)	≥ 35	50.0	53.3	0.27	Low

Optimal cut-offs were determined by Youden's J maximization. Bold-faced text is used for (i) column headers, (ii) tier classifications, and (iii) biomarker names in the first column. The meaning of all bold-faced terms is provided below.

(i) Column headers (bold): Marker — host immunological analyte assessed; AUC (95% CI) — area under the ROC curve with 95% confidence interval; Cut-off — optimal discriminatory threshold value (in marker-specific units) determined by Youden's J statistic that maximizes the sum of sensitivity and specificity; Sens (%) — sensitivity (true-positive rate, %); Spec (%) — specificity (true-negative rate, %); p — two-tailed p-value for the AUC differing from 0.50 (no discrimination); Tier — diagnostic-performance category (see below).

(ii) Tier classifications (bold): High — AUC ≥ 0.80 (strong discriminatory performance, considered clinically informative); Moderate — 0.70 ≤ AUC < 0.80 (acceptable performance, potential adjunctive value); Low — AUC < 0.70 (insufficient discrimination; not recommended as a stand-alone marker).

(iii) Biomarker names (bold, first column): Markers were measured at baseline as described in Section 2.3.

Lymphocyte subpopulations (flow cytometry): CD3⁺, CD3-positive T-lymphocytes; CD4⁺, CD4-positive T-helper lymphocytes; CD4/CD8 ratio, ratio of CD4⁺ to CD8⁺ T-lymphocytes; CD16⁺, CD16-positive natural killer cells; CD20⁺, CD20-positive B-lymphocytes; CD25⁺, CD25-positive (IL-2Rα) activated lymphocytes; CD95⁺, CD95-positive (Fas/APO-1) lymphocytes.

Serum cytokines (ELISA): IL-1β, interleukin-1 beta; IL-4, interleukin-4; IL-6, interleukin-6; TNF-α, tumor necrosis factor alpha; IFN-γ, interferon gamma; IP-10, interferon-γ-induced protein 10 (CXCL10).

Endothelial activation markers: sICAM-1, soluble intercellular adhesion molecule-1; sVCAM-1, soluble vascular cell adhesion molecule-1.

Fibrinolytic markers: tPA, tissue plasminogen activator; PAI-1, plasminogen activator inhibitor-1.

Immunoglobulins: IgA, immunoglobulin A; IgG, immunoglobulin G; IgM, immunoglobulin M.

IgG showed moderate discrimination only (AUC 0.79; p = 0.002). All remaining markers — CD3^+^, CD4^+^, CD16^+^, CD20^+^, and CD25^+^ percentages; IL-1β and IL-4; IgA and IgM; and tPA — fell into the low-discrimination tier (AUC 0.56–0.66) and did not reach statistical significance individually (p > 0.05).

The nine high-AUC markers map onto three coherent pathophysiological axes — endothelial-fibrinolytic (sICAM-1, sVCAM-1, PAI-1), pro-inflammatory cytokine (IL-6, TNF-α), and Th1/cellular with apoptotic priming (CD4/CD8, IFN-γ, IP-10, CD95^+^) — matching the network structure identified in the correlation analysis.

## 4 Discussion

In a prospective cohort of 115 adults with confirmed OATB, nine key host markers achieved high prognostic differentiation for complicated course (AUC ≥ 0.80), along three linked pathophysiological axes: endothelial-fibrinolytic (sICAM-1, sVCAM-1, PAI-1), pro-inflammatory cytokine (IL-6, TNF-α), and apoptotic-priming Th1/cell (CD4/CD8, IFN-γ, IP-10, CD95^+^). The dominance of endothelial markers - sICAM-1 (AUC 0.89) and sVCAM-1 (0.87) ranked first and third, respectively, extends previous observations limited to pulmonary tuberculosis (Jumaar et al., 2025; Mitroi et al., 2025) to osteoarticular conditions and suggests that microvascular dysfunction may be a central, rather than peripheral, feature of complicated OATB. The strong correlation between pro-inflammatory cytokines and endothelial activation markers in the complex subgroup (r = 0.42–0.56) supports a self-reinforcing cycle in which TNF-α-driven endothelial activation promotes PAI-1 release and fibrinolytic suppression, leading to tissue hypoxia that further enhances inflammatory signaling (Esmon, 2005; Levi & van der Poll, 2017).

The findings of the Th1/cell axis decreased CD4/CD8 ratio, suppressed IFN-γ and IP-10, and elevated CD95^+^ are consistent with the paradigm of Th1 exhaustion and apoptotic priming in chronic mycobacterial infection (Sia & Rengarajan, 2019; Zhuang et al., 2024). The convergence of these three axes into a plausible interconnected pattern distinguishes complicated and uncomplicated OATB and provides a biological basis for multi-marker prognostic panels.

These findings should be interpreted within the context of several important limitations. The ROC analysis was based on a prognostic subgroup of 30 patients, yielding wide 95% confidence intervals and increasing the risk of optimistic AUC estimates. The single-center design from a single region of Uzbekistan limits generalizability; external validation in independent multicenter cohorts, including patients coinfected with multidrug-resistant TB and HIV is essential. The assessment of outcomes was performed by a multidisciplinary panel, operationally separated from laboratory data, but no formal inter-rater reliability testing was performed. The complicated group showed a more advanced histomorphological stage and higher baseline ESR and CRP (**Table 1**), raising the possibility that immunological differences reflect, in part, disease severity rather than independent prognostic biology; Multivariate adjustment for disease stage is warranted in future studies. We did not formally combine markers into a composite score, although the strong correlation suggests that parsimonious indices may achieve high accuracy. Finally, the observational design cannot establish causality.

If externally validated, the nine candidate prognostic markers may contribute to risk stratification in newly diagnosed OATB. Whether such stratification influences clinical decision-making, including timing of surgical evaluation or intensity of radiological monitoring, remains a hypothesis that requires prospective study.

## 5 Conclusion

In a prospective cohort of 115 adults with confirmed osteoarticular tuberculosis, a baseline 20-marker host immunological panel identified nine markers with high prognostic discrimination for complicated disease course (AUC ≥ 0.80, p < 0.01): sICAM-1, sVCAM-1, IL-6, CD4/CD8 ratio, IP-10, TNF-α, CD95^+^, IFN-γ, and PAI-1. These nine markers map onto three coupled pathophysiological axes — endothelial-fibrinolytic, pro-inflammatory cytokine, and Th1/cellular with apoptotic priming that the correlation network identified independently as the structural backbone of immune dysregulation in OATB. The endothelial-fibrinolytic axis, largely unstudied in OATB to date, contributed the highest individual AUC values, supporting the hypothesis that microvascular activation is a potentially important, measurable contributor to tissue destruction in this form of extrapulmonary tuberculosis. Within this framework, the panel is best understood not as nine independent biomarkers but as three coupled readouts of a single transition from immune competence to functional immune insufficiency.

All nine markers are measured using routine flow cytometry and ELISA platforms, with clinically practical Youden-J-derived cutoffs (sensitivity 76.7–86.7%; specificity 73.3–83.3%). If validated in independent cohorts, a single baseline blood draw before the start of anti-TB chemotherapy may contribute to risk stratification in newly diagnosed OATB. Whether such stratification influences clinical decision-making, including timing of surgical evaluation or intensity of radiological monitoring, remains a hypothesis that requires prospective study. External validation in independent multicenter cohorts, including patients co-infected with multidrug-resistant TB and HIV is an important next step.

## Data Availability

The original contributions presented in the study are included in the article/supplementary material. Further inquiries can be directed to the corresponding author.
